# Supplementary material for: Time-series transcriptome analysis identified differentially expressed genes in broiler chicken infected with mixed Eimeria species
Source: Front Genet. 2022 Aug 8;13:886781. doi: 10.3389/fgene.2022.886781 (PMC9393255; doi:10.3389/fgene.2022.886781)
Supplement: Supplementary file 2 [file DataSheet1.ZIP › 4dpi_KEGG.Gsea.1625100298584/KEGG_OXIDATIVE_PHOSPHORYLATION.html]

Details for gene set KEGG\_OXIDATIVE\_PHOSPHORYLATION[GSEA]

|  || Dataset | TMM\_4dpi\_gct\_format\_4dpi\_gct\_format.Class\_4dpi.cls #PC\_versus\_NC.Class\_4dpi.cls #PC\_versus\_NC\_repos |
| Phenotype | Class\_4dpi.cls#PC\_versus\_NC\_repos |
| Upregulated in class | 0 |
| GeneSet | KEGG\_OXIDATIVE\_PHOSPHORYLATION |
| Enrichment Score (ES) | -0.43348548 |
| Normalized Enrichment Score (NES) | -1.7654355 |
| Nominal p-value | 0.002072539 |
| FDR q-value | 0.089653775 |
| FWER p-Value | 0.2444 |
Table: GSEA Results Summary

  

Fig 1: Enrichment plot: KEGG\_OXIDATIVE\_PHOSPHORYLATION      
 Profile of the Running ES Score & Positions of GeneSet Members on the Rank Ordered List

  

| SYMBOL | TITLE | RANK IN GENE LIST | RANK METRIC SCORE | RUNNING ES | CORE ENRICHMENT || 1 | NDUFA5 | na | 2366 | 0.367 | -0.1783 | No |
| 2 | ATP6V1C2 | na | 2703 | 0.323 | -0.1889 | No |
| 3 | NDUFS4 | na | 3257 | 0.249 | -0.2217 | No |
| 4 | NDUFB3 | na | 3454 | 0.226 | -0.2257 | No |
| 5 | UQCR10 | na | 3517 | 0.218 | -0.2190 | No |
| 6 | ATP6V1G3 | na | 4013 | 0.166 | -0.2514 | No |
| 7 | ATP6V0A4 | na | 4081 | 0.161 | -0.2483 | No |
| 8 | PPA2 | na | 4319 | 0.140 | -0.2605 | No |
| 9 | NDUFS2 | na | 4445 | 0.129 | -0.2639 | No |
| 10 | NDUFA6 | na | 4773 | 0.100 | -0.2859 | No |
| 11 | ATP6V1D | na | 4796 | 0.098 | -0.2824 | No |
| 12 | NDUFS8 | na | 4843 | 0.094 | -0.2811 | No |
| 13 | ATP6V0D2 | na | 4847 | 0.094 | -0.2763 | No |
| 14 | NDUFA4 | na | 4930 | 0.086 | -0.2785 | No |
| 15 | SDHC | na | 4978 | 0.080 | -0.2780 | No |
| 16 | TCIRG1 | na | 5120 | 0.069 | -0.2861 | No |
| 17 | ATP6V1E1 | na | 5343 | 0.050 | -0.3020 | No |
| 18 | NDUFB9 | na | 5854 | 0.005 | -0.3445 | No |
| 19 | ATP6V1H | na | 5862 | 0.005 | -0.3448 | No |
| 20 | NDUFB1 | na | 5911 | 0.000 | -0.3488 | No |
| 21 | NDUFV3 | na | 5984 | -0.005 | -0.3546 | No |
| 22 | UQCRB | na | 6027 | -0.008 | -0.3576 | No |
| 23 | UQCRFS1 | na | 6104 | -0.015 | -0.3632 | No |
| 24 | NDUFB7 | na | 6184 | -0.021 | -0.3687 | No |
| 25 | ATP6V0E1 | na | 6287 | -0.029 | -0.3756 | No |
| 26 | COX7C | na | 6335 | -0.033 | -0.3778 | No |
| 27 | ATP6V0D1 | na | 6337 | -0.033 | -0.3761 | No |
| 28 | ATP6V0C | na | 6344 | -0.033 | -0.3748 | No |
| 29 | NDUFC2 | na | 6428 | -0.041 | -0.3795 | No |
| 30 | NDUFA1 | na | 6469 | -0.044 | -0.3805 | No |
| 31 | NDUFA11 | na | 6628 | -0.057 | -0.3906 | No |
| 32 | SDHA | na | 6633 | -0.057 | -0.3879 | No |
| 33 | NDUFS1 | na | 6746 | -0.066 | -0.3936 | No |
| 34 | PPA1 | na | 7064 | -0.095 | -0.4150 | No |
| 35 | UQCRC1 | na | 7129 | -0.101 | -0.4149 | No |
| 36 | COX7B | na | 7211 | -0.110 | -0.4157 | No |
| 37 | ATP6V0A2 | na | 7233 | -0.112 | -0.4113 | No |
| 38 | NDUFV2 | na | 7239 | -0.112 | -0.4056 | No |
| 39 | NDUFA2 | na | 7492 | -0.135 | -0.4194 | No |
| 40 | NDUFA7 | na | 7501 | -0.135 | -0.4127 | No |
| 41 | NDUFB6 | na | 7750 | -0.157 | -0.4249 | Yes |
| 42 | UQCRC2 | na | 7779 | -0.159 | -0.4185 | Yes |
| 43 | NDUFA10 | na | 7839 | -0.165 | -0.4145 | Yes |
| 44 | NDUFB5 | na | 7888 | -0.169 | -0.4093 | Yes |
| 45 | COX6A1 | na | 7900 | -0.170 | -0.4009 | Yes |
| 46 | UQCRHL | na | 7905 | -0.171 | -0.3919 | Yes |
| 47 | NDUFS3 | na | 7925 | -0.172 | -0.3841 | Yes |
| 48 | NDUFB8 | na | 7975 | -0.177 | -0.3786 | Yes |
| 49 | SDHD | na | 8030 | -0.182 | -0.3732 | Yes |
| 50 | NDUFA9 | na | 8119 | -0.191 | -0.3701 | Yes |
| 51 | ATP12A | na | 8132 | -0.191 | -0.3607 | Yes |
| 52 | NDUFS6 | na | 8204 | -0.198 | -0.3558 | Yes |
| 53 | NDUFS7 | na | 8226 | -0.201 | -0.3466 | Yes |
| 54 | ATP6V0A1 | na | 8243 | -0.203 | -0.3369 | Yes |
| 55 | ATP6V1C1 | na | 8282 | -0.207 | -0.3288 | Yes |
| 56 | ATP6V1A | na | 8482 | -0.227 | -0.3331 | Yes |
| 57 | COX17 | na | 8517 | -0.231 | -0.3233 | Yes |
| 58 | NDUFA8 | na | 8530 | -0.233 | -0.3116 | Yes |
| 59 | COX15 | na | 8891 | -0.272 | -0.3270 | Yes |
| 60 | ATP6V1G1 | na | 8935 | -0.277 | -0.3154 | Yes |
| 61 | NDUFAB1 | na | 9185 | -0.306 | -0.3196 | Yes |
| 62 | NDUFB4 | na | 9213 | -0.310 | -0.3049 | Yes |
| 63 | NDUFV1 | na | 9261 | -0.316 | -0.2916 | Yes |
| 64 | COX7A2 | na | 9263 | -0.316 | -0.2744 | Yes |
| 65 | NDUFB2 | na | 9490 | -0.347 | -0.2744 | Yes |
| 66 | NDUFS5 | na | 9588 | -0.361 | -0.2629 | Yes |
| 67 | COX6C | na | 9667 | -0.370 | -0.2492 | Yes |
| 68 | UQCRQ | na | 9806 | -0.387 | -0.2397 | Yes |
| 69 | LHPP | na | 9847 | -0.392 | -0.2217 | Yes |
| 70 | UQCR11 | na | 9903 | -0.399 | -0.2045 | Yes |
| 71 | COX5A | na | 9946 | -0.406 | -0.1859 | Yes |
| 72 | NDUFB10 | na | 10015 | -0.417 | -0.1688 | Yes |
| 73 | ATP5PB | na | 10163 | -0.439 | -0.1572 | Yes |
| 74 | COX10 | na | 10242 | -0.451 | -0.1391 | Yes |
| 75 | ATP6V1B2 | na | 10256 | -0.454 | -0.1155 | Yes |
| 76 | ATP6V0B | na | 10748 | -0.548 | -0.1267 | Yes |
| 77 | COX7A2L | na | 10772 | -0.553 | -0.0985 | Yes |
| 78 | COX4I1 | na | 10859 | -0.575 | -0.0743 | Yes |
| 79 | COX11 | na | 10912 | -0.585 | -0.0468 | Yes |
| 80 | ATP6V0E2 | na | 11169 | -0.654 | -0.0325 | Yes |
| 81 | ATP6AP1 | na | 11499 | -0.807 | -0.0161 | Yes |
| 82 | SDHB | na | 11792 | -1.078 | 0.0183 | Yes |
Table: GSEA details [plain text format]

  

Fig 2: KEGG\_OXIDATIVE\_PHOSPHORYLATION      
 Blue-Pink O' Gram in the Space of the Analyzed GeneSet

  

Fig 3: KEGG\_OXIDATIVE\_PHOSPHORYLATION: Random ES distribution      
 Gene set null distribution of ES for **KEGG\_OXIDATIVE\_PHOSPHORYLATION**
